# Huangqi Jianzhong Tang for Treatment of Chronic Gastritis: A Systematic Review of Randomized Clinical Trials

**DOI:** 10.1155/2015/878164

**Published:** 2015-12-27

**Authors:** Yue Wei, Li-Xin Ma, Sheng-Jun Yin, Jing An, Qi Wei, Jin-Xiang Yang

**Affiliations:** ^1^The Third Affiliated Hospital of Beijing University of Chinese Medicine, 51 An Wai Xiaoguan Street, Chaoyang District, Beijing 100029, China; ^2^Hebei University, Baoding, Hebei, China; ^3^The Second Hospital of Tianjin Medical University, Tianjin, China; ^4^General Forestry Station of Beijing Municipality, Beijing, China

## Abstract

To assess the clinical effects and safety of Huangqi Jianzhong Tang (HQJZ) for the treatment of chronic gastritis (CG), three English databases and four Chinese databases were searched through the inception to January 2015. In randomized controlled trials (RCTs) comparing HQJZ with placebo, no intervention and western medicine were included. A total of 9 RCTs involving 979 participants were identified. The methodological quality of the included trials was generally poor. Meta-analyses demonstrated that HQJZ plus conventional medicine was more effective in improving overall gastroscopy outcome than western medicine alone for treatment of chronic superficial gastritis with the pooling result of overall improvement [OR 3.78 (1.29,11.06), *P* = 0.02]. In addition, the combination of HQJZ with antibiotics has higher overall effect rate than antibiotics alone for the treatment of CG [OR 2.60 (1.49,4.54), *P* = 0.0007]. There were no serious adverse events reported in both the intervention and controlled groups. HQJZ has the potential of improvement of the patients' gastroscopy outcomes,* Helicobacter pylori* clearance rate, traditional Chinese Medicine syndromes, and overall effect rate alone or in combination use with conventional western medicine for chronic atrophic gastritis. However, due to poor methodological quality, the beneficial effect and safeties of HQJZ for CG could not be confirmed.

## 1. Introduction

Chronic gastritis (CG) is defined as chronic inflammatory cells infiltration in gastric mucosa [1]. They are classified into chronic nonatrophic gastritis (CSG) and chronic atrophic gastritis (CAG) based on the endoscopic appearances and histopathologic patterns of the gastric mucosa.* Helicobacter pylori* (Hp) infection in the stomach lining is the most common and likely causes, leading to some gastric glandular cells which can be lost and eventually replaced by intestinal and fibrous tissues or even worse associated with gastric cancer during their long process of the disease [1, 2].

CG is a kind of the most common digestive system diseases in clinical practice, with estimated 50% of the world population having the Hp infection [3, 4]. And there is a lack of effective drug for CG with about 20% recurrence rate [5]. Huangqi Jianzhong Tang (HQJZ), a traditional Chinese Medicine (TCM), is commonly used for treatment of CG in China. Here a systematic review and meta-analysis of randomized controlled trials were conducted to evaluate its therapeutic effects on the treatments of CG patients.

## 2. Materials and Methods

### 2.1. Searching Strategy

Two authors (Yue Wei and Li-Xin Ma) identified the citations by searching three English electronic databases (PubMed, Embase, and Cochrane Library) and four Chinese electronic databases (China National Knowledge Infrastructure (CNKI), Chinese Biomedicine (SinoMed), Chinese Scientific Journals Database (VIP), and Wanfang database) from their inception through January 2015. Conference proceedings and dissertations were also searched from CNKI and Wanfang databases for unpublished trials. Searching strategies were made through the way of text word, key words, and MeSH terms. The following terms (Chinese equivalent) were used individually or in combination with each other including “atrophic gastritis”, “chronic atrophic gastritis”, “chronic gastritis”, “chronic”, “atrophic”, “gastritis”, “precancerous lesions of gastric cancer”, “intestinal metaplasia”, “dysplasia”, “Chronic superficial gastritis”, “superficial gastritis”, “chronic non atrophic gastritis”, “non atrophic gastritis”, “huangqi jianzhong Formula”, “huangqi jianzhong decoction”, “huangqi jianzhong tang”, “huangqi jianzhong capsules”, “huangqi jianzhong pills”, “huangqi jianzhong tablets”, and “random”. There is no restriction for publication language and time. We retrieved the titles and abstract using the reference management software NoteExpress V 3.0.

### 2.2. Inclusion/Exclusion Criteria

*Types of Studies.* Randomized controlled trials were included, as well as crossover randomized trials, but only the outcomes from the first period of treatment were extracted and analyzed. Quasi-randomized trials were excluded. Two authors screened the titles and abstracts by eliminating the duplications, animal test, and other mechanical studies. Then the full articles were retrieved and the relevant studies were included. The disagreements were settled by consulting the third author.

*Types of Participants.* The participants diagnosed with CG (containing CAG and CSG) by gastroscopy and pathology were included. There are no limitations for the age, sex, and comorbidities.

*Types of Interventions.* The patients in the experiment group were orally administered HQJZ, which were in any preparations such as pills, capsules, decoctions, and tablets. Treatment course was more than 2 weeks. Modified HQJZ changes based on TCM syndrome differentiations and treatment variations were acceptable. The controlled group could be placebo, with no intervention and western medicine. The trials of intervention of HQJZ ± western medicine ± supportive treatment were included.

*Types of Outcome Measures.* The primary outcome was the improvement of atrophy and intestinal metaplasia based on the gastroscopy and pathology, and the incidence of gastric cancer. The secondary outcome was the score of TCM syndromes, the clinical symptom improvement rate (stomachache, gastrectasia, dyspepsia, shapeless stools, etc.), quality of life (QOL), Hp clearance rate, and overall effect rate.

### 2.3. Assessment of Risk of Bias

Two authors (Yue Wei and Li-Xin Ma) independently assessed the quality of included trials using the Cochrane risk of bias table [6]. The following items were assessed: random sequence generation, allocation concealment, blinding, incomplete outcome data, selective outcome reporting, and other bias. In addition, estimation of sample size and consistency of the baseline characteristic were also considered for the assessment of the bias. Disagreements were resolved by discussion with a third author (Jin-Xiang Yang). The risk of bias was categorized as low, unclear, or high.

### 2.4. Data Analysis

Two reviewers independently conducted the screening of studies, and data extraction (Yue Wei and Li-Xin Ma). Epidata 3.1 was used for data extraction. Meta-analyses were performed using RevMan 5.2 software. We pooled data using odds ratio (OR) with 95% confidence interval (CI) for dichotomous outcomes or mean difference (MD) with 95% CI for continuous outcomes. If different measurement scales were used, standardized mean differences (SMD) were analyzed. For crossover trials, only the outcomes from the first period were included. Where data were not reported, the data was requested from the corresponding author. A fixed effects model was used unless there was evidence of heterogeneity. Heterogeneity was assessed by the chi-squared test and/or the *I*-squared statistic. The *α* ≤ 0.1 and/or *I* ^2^ ≥ 45% was indicative of substantial heterogeneity. When heterogeneity was present, subgroup analysis and sensitivity analysis were conducted to evaluate the robustness of the results. Funnel plots were performed to detect publication bias.

## 3. Results

### 3.1. Description of Studies

**Figure 1:**
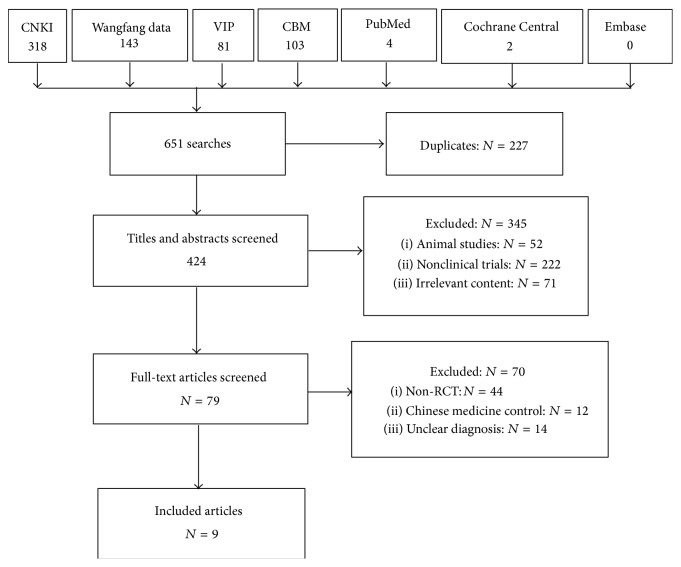
Flow chart of literature search.

The searching flow chart is presented in Figure 1. There were 9 randomized clinical trials (RCTs) (*N* = 979) in this systematic review. All RCTs were conducted in China and all studies published in full in Chinese. There was no multicentre trial. Two studies [7, 8] were conducted to evaluate the effects of HQJZ for the treatment of CAG. Three studies [9–11] assessed the effects of HQJZ for the treatment of CSG. And four trials [12–16] explored the effects of HQJZ for the treatment of CG. The sample size was from 60 [8] to 238 [13]. Participants are from 19 to 83 years old. The disease courses were from 1.5 months to 27 years, except for 1 trial [11] that did not mention the clinical course. Five trials reported the TCM syndrome differentiation and treatment variation, of which 4 trials [8, 9, 12, 13] reported the participants' syndrome of deficiency cold in spleen and stomach, and in another 1 trial [7], the participants have the syndrome of weakness in spleen and stomach. Almost all trials included reported that HQJZ was applied in the intervention group; only 1 trial [8] used its modified formulas. The courses of treatment were from 2 weeks [12] to 3 months [7].

The comparisons included the following: HQJZ versus western drugs (6 trials) [7, 8, 10–12, 14] and HQJZ + western drugs versus western drugs (3 trials) [9, 13, 15].

As for primary outcome reporting, seven trials reported the results of gastroscopy and pathology [7–12, 15] and two of them also reported the cure rate of Hp infection [11, 15]. While two trials did not report the pathology results [13, 14], one of the trials reported clinical symptoms and signs only [14]. In addition, 3 trials [7–9] reported the improvement effect in TCM syndromes.  Table 1 lists the characteristics of the studies including interventions used in the control and treatment groups, outcomes, and methodological qualities.

### 3.2. Risk of Bias Assessment

Only three of the 9 trials (33.3%) described how subjects were randomly assigned into the intervention group and the controlled group. They all used a random number table [8, 9, 12]. The remaining six trials (66.7%) simply mentioned “randomization” but did not report the specific method.

None of the trials mentioned the allocation concealment and blindness. In addition, no trial reported their estimation of sample size, flow chart of the trial, and the utilization of intention-to-treat analysis. There was neither any information about trial registration nor incomplete outcome reporting. The risk bias assessment of the methodological quality lists is shown in Table 2.

### 3.3. Clinical Effect

#### 3.3.1. Improvement of Atrophy and Intestinal Metaplasia under the Gastroscopy Pathology

*HQJZ + Western Medicine versus Western Medicine*. One trial reported the effect rate of overall improvement and pathology changes under the gastroscopy for patients with CSG [9]. Study showed that there were statistically significant differences for gastroscopy improvement rate [OR 3.78 (1.29,11.06), *P* = 0.02] and pathology improvement rate [OR 2.83 (1.00,7.98), *P* = 0.05] between the comparisons of HQJZ ± western medicine groups. See Table 2.

#### 3.3.2. Incidence of Gastric Cancer, Clinical Symptom Improvement, and QOL

Our review did not find any assessment on the effects of incidence of gastric cancer, clinical symptom improvement rate, or QOL of HQJZ for patients with CG, CSG, or CAG among the included trials.

#### 3.3.3. Improvement of TCM Syndromes

*HQJZ versus Western Medicine*. For the improvement of TCM syndrome effect of treatment on the patients with CAG [7, 8], meta-analysis showed that there was a statistically significant difference for the comparison between HQJZ and domperidone + vatacoenayme ([OR 6.67 (1.41,31.59), *P* = 0.02]) [7] or HQJZ and domperidone [MD −5.85, (−7.71, −3.99), *P* < 0.00001] [8]. See Table 2.

*HQJZ + Western Medicine versus Western Medicine*. Only one trial reported the effects of TCM syndromes for the combination use of western medicine ± HQJZ for patients with CSG [9]. Study results showed statistically significant difference between the two groups [OR 9.75 (1.16,82.11), *P* = 0.04]. See Table 2.

#### 3.3.4. Hp Clearance Rate

*HQJZ + Western Medicine versus Western Medicine.* We included one trial on the effects of Hp clearance rate for patients with CSG [9]. There was statistically significant difference between the comparisons of HQJZ ± western medicine (omeprazole and domperidone) groups [OR 6.02 (1.43,25.40), *P* = 0.01]. See Table 2.

#### 3.3.5. Overall Effect Rate

**Figure 2:**
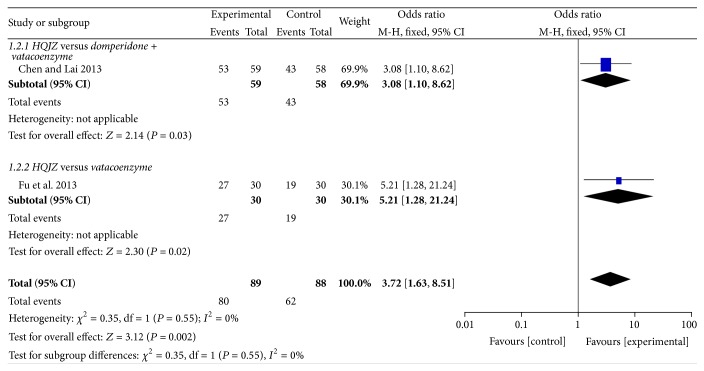
Forest plot of improvement of overall effect rate for patients with CAG.

*HQJZ versus Western Medicine.* For the treatment of the patients with CAG [7, 8], two studies comparing the overall effects between HQJZ and domperidone or vatacoenayme were included in the pooling results. There was a statistically significant overall effect rate comparing HQJZ and western medicine [OR 3.72, 95% CI (1.63,8.51), *P* = 0.002]. See Figure 2.

**Figure 3:**
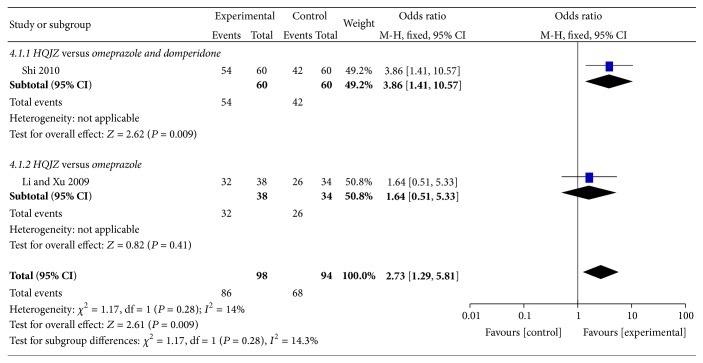
Forest plot of improvement of overall effect rate for patients with CSG.

For the treatment of the patients with CSG [10, 11], two studies comparing the overall effects between HQJZ and omeprazole or domperidone were included in the pooling results. There was a statistically significant overall effect rate comparing HQJZ and western medicine [OR 2.73 (1.29,5.81), *P* = 0.009]. See Figure 3.

We included one study comparing the overall effect rate between HQJZ and omeprazole for the treatment of the patients with CG (not classified as atrophic and nonatrophic) [14]. There was no statistically significant difference between HQJZ and omeprazole group [OR 3.62 (0.90,14.63), *P* = 0.07 > 0.05].

*HQJZ + Western Medicine versus Western Medicine.* For the treatment of the patients with CSG [9], we included one trial comparing the overall effect rate of the combination of HQJZ plus omeprazole + domperidone with the omeprazole + domperidone. There was no statistically significant difference between the two groups [OR 3.58 (0.89,14.39), *P* = 0.07 > 0.05].

**Figure 4:**
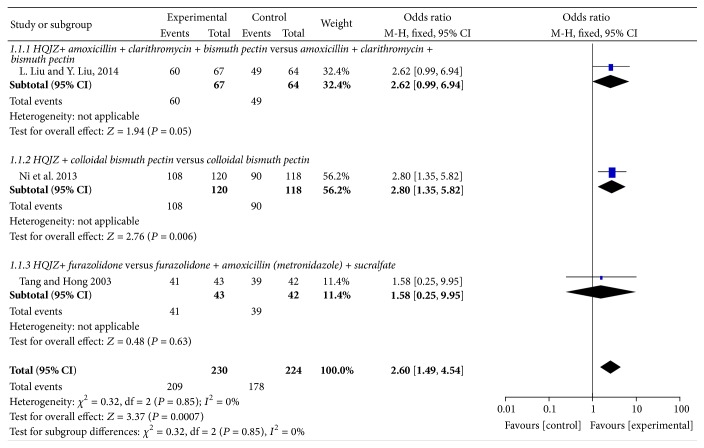
Forest plot of improvement of overall effect rate for patients with CG.

There were three trials comparing the overall effect rate of combined intervention of western medicine ± HQJZ for patients with CG [12, 13, 15]. A statistically significant difference between the comparing groups was found [OR 2.60 (1.49,4.54), *P* = 0.0007]. See Figure 4.

*HQJZ plus Colloidal Bismuth Pectin versus Colloidal Bismuth Pectin.* Results showed that there was statistically significant difference between the intervention group of HQJZ plus colloidal bismuth pectin and the controlled group of colloidal bismuth pectin [OR 2.80 (1.35,5.82), *P* = 0.006] [13].

*HQJZ plus Clarithromycin + Amoxicillin + Bismuth Pectin versus Clarithromycin + Amoxicillin + Bismuth Pectin. *There was no statistically significant difference between the two groups of clarithromycin, amoxicillin, and bismuth pectin ± HQJZ [OR 2.62 (0.99,6.94), *P* = 0.05] [12].

*HQJZ + Furazolidone versus Furazolidone + Amoxicillin (Metronidazole) + Sucralfate.* There was no statistically significant difference between the two groups of furazolidone, amoxicillin (or metronidazole if allergy), and sucralfate, ±HQJZ [OR 1.58 (0.25,9.95), *P* = 0.63 > 0.05] [15].

#### 3.3.6. Adverse Reaction

Five of the 9 trials mentioned adverse effects [8, 9, 13–15]. Two of them reported that there was not any adverse effect observed in HQJZ application [8, 9]. One trial [13] reported the adverse effects including rash and anaphylactoid purpura found in legs for 1 case and increase of eosinophils cell count in the blood test for 1 case in the controlled group and with no adverse effect found in intervention group. Another one trial [14] mentioned that the clinical symptoms of epigastric pain, fullness, belching, and poor appetite were observed both in intervention and in controlled groups, relatively minor in the intervention group than those in the controlled group. The other trial [15] reported that most of patients had poor appetite, upper abdominal discomfort, nausea and vomiting, and so forth in the control group especially for those patients who were given metronidazole, metoclopramide, and anisodamine (see Table 2).

## 4. Discussion

### 4.1. About HQJZ

The prescription ofHQJZ is made of seven Chinese herbal drugs including* astragalus*,* cassia twig*,* white peony root*,* baked licorice*,* ginger*,* jujube*, and* maltose*. Reports on the effects of HQJZ for the treatment of patients with CG came from a TCM classic named* Synopsis of Golden Chamber*, written by Zhang Zhongjing, and dated back to more than 1800 years before (Eastern Han Dynasty of China) [16]. It has been used in the clinical scenario of patients with the abdominal upset or pain, with or without belching, abdominal bloating, nausea, vomiting, and loose stools or a feeling of fullness, of burning in the upper abdomen, or of cold and weakness in the limb. Up to now there are some evidences reporting its mechanism in the treatment of CG. Evidence from an animal test in rat models with spleen-asthenia showed that the HQJZ might regulate serum gastrin levels and significantly inhibit pepsinogen secretion of the chief cells and the acid secretion of the oxyntic mucosa [17]. Another experiment showed that the HQJZ could elevate the levels of substance P in gastric antrum and facilitates gastric emptying [18]. The results of the third experimental test demonstrated that HQJZ might set in motion mechanisms involving the improvement of energy metabolism in colonic mucosal injury induced by 2,4,6-trinitrobenzene sulfonic acid (TNBS) [19]. Evidences from the clinical trials also found that HQJZ may reduce fatigue by increasing the oxygen uptake and the systemic utility of oxygen among twelve senior male high school basketball players [20]. In one word, HQJZ may be a multitargeting management for the treatment of patients with CG.

### 4.2. Main Findings

9 RCTs and 979 participants were included in this review. Firstly our meta-analysis of the overall effect rate found that HQJZ ± western medicine were more effective than western medicine for the treatment of CG. Secondly, HQJZ was more effective in improving the symptoms and signs than western medicine for patients with CAG; and these effects were also found when comparing the groups of HQJZ ± western medicine for the treatment of patients with CSG. Thirdly, studies showed that HQJZ plus western medicine had more effects on increasing Hp clearance rate and improving gastroscopic manifestation than western medicine for treatment of CSG.

### 4.3. Limitations of This Review

The following are some limitations existing in the included RCTs:

1. The included studies had limitations in methodological qualities. Only 3 of the trials reported on how the participants are randomly assigned to the intervention groups. Six out of 9 trials (66.7%) simply mentioned “randomization,” with none of the trials mentioning the use of allocation concealment, and blinding. 5 of the 9 trials mentioned adverse reaction. None of the trials mentioned follow-up.
2. Although Hp infection is the most frequent cause of CG, there are many other causes of gastritis [21, 22]. In this review only 2 of the 9 trials made a clear statement in including patients diagnosed with CAG. Four studies did not classify CG into subtype of CSG and CAG based on pathology test. Instead, 9 RCTs reported TCM syndrome diagnosis, such as* deficiency and cold of spleen and stomach*, resulting in lower external validity and impaired clinical application of the results under these circumstances.
3. CG has relatively minor manifestation in the process of the diseases. And no universally accepted classification system provides an entirely satisfactory description of all of the gastritis and gastropathies [23]. So there is a need to report explicitly the endoscopic appearances and histopathologic patterns of the gastric mucosa tests in RCTs. However only 1 of the trials reported gastroscopy and pathology separately. 2 of them did not contain pathologic outcome reporting. In addition, most of the trials even used the overall effect rate as the main outcome; this will lead to failure to quantitatively assess the effectiveness of HQJZ on the treatment of patients with CG.

## 5. Conclusions

HQJZ may have potential effects on the treatment of patients with CG. However, due to limitation of the methodological quality, we could not draw confirmed conclusion on its beneficial effect as well as its risks. Future clinical trials on evaluating the effects of HQJZ should be designed more rigorously in methodological quality.

## Figures and Tables

**Table 1:** An overview of the included studies.

Study ID	Age (years)	Classification of chronic gastritis	Type of syndrome	Course of disease (years)	Male (%)	Sample size *N* (*n*/*n*)	Intervention	Control medicine	Time of treatment (weeks)	Outcome measures
Chen and Lai 2013 [7]	21–62	Chronic atrophic gastritis	Weakness of spleen and stomach	1–14	58	117 (59/58)	HQJZ (and stagnation, added Costas, *Amomum villosum*, and blood stasis, added *Salvia miltiorrhiza*, *Panax notoginseng*, and yin-deficiency, added *Polygonatum*, dwarf lilyturf, and indigestion, added Jiaosanxian, and damp-heat, added *Coptis chinensis*, and cold-dampness, added *Atractylodes*)	Domperidone 10 mg tid, vatacoenayme 1 g tid	12	Overall effect (clinical symptoms, signs, manifestations of gastroscopy, and pathology), TCM syndrome effect (TCM symptoms and signs)

Fu et al. 2013 [8]	30–70	Chronic atrophic gastritis	Deficiency cold of spleen and stomach	1–10	53	60 (30/30)	HQJZ	Vatacoenayme 1 g tid	8	Overall effect (clinical symptoms, signs, manifestations of gastroscopy, and pathology), TCM syndrome effect (TCM symptoms and signs)

Zhang 2013 [9]	19–65	Chronic nonatrophic gastritis	Deficiency cold of spleen and stomach	1–11	56	80 (40/40)	HQJZ + western medicine (and loose stools, added parched hyacinth bean 15 g, *Coix* seed 15 g, and fullness, added citron 10 g, *Magnolia* 10 g, and stomachache, added Rhizoma *Corydalis* 10 g, and weakness, added red ginseng 10 g, and loss of appetite, added Jiaosanxian 15 g, *Amomum villosum* 6 g, and vomiting, added *Pinellia* 10 g, and acid regurgitation, heartburn, added Cuttlebone 18 g, fritillary bulb 15 g)	Omeprazole 20 mg bid for 4 weeks, or +domperidone 10 mg tid for 4 weeks, or +amoxicillin 0.5 g bid, metronidazole 0.4 g bid for 1 week	4	Overall effect (clinical symptoms, signs, manifestations of gastroscopy, and pathology), TCM syndrome effect (TCM symptoms and signs), Hp clearance, gastroscope, and pathology

Shi 2010 [10]	19–65	Chronic nonatrophic gastritis		1–27	55	120 (60/60)	HQJZ (added *Bupleurum* 10 g, Radix Aucklandiae 10 g)	Omeprazole 40 mg qd, domperidone 10 mg tid	4	Overall effect (clinical symptoms, signs, manifestations of gastroscopy, and pathology)

Li and Xu 2009 [11]	18–63	Chronic nonatrophic gastritis			47	72 (38/30)	HQJZ (added *Evodia rutaecarpa* 10 g)	Omeprazole 20 mg qd	4	Overall effect (clinical symptoms, signs, manifestations of gastroscopy, and pathology, Hp)

L. Liu and Y. Liu 2014 [12]	30–76	Chronic gastritis	Deficiency cold of spleen and stomach	0.25–11	56	131 (67/64)	HQJZ + control medicine (added *lanceolata* 20 g, *Atractylodes* 15 g, *Poria cocos *30 g, Tangerine Peel 8 g, *Pinellia* 10 g, bitter orange 15 g, corium stomachium galli 15 g, *Salvia* 15 g, *Panax notoginseng* powder 4 g, and Cuttlebone 8 g, and stomach fullness, added Radix Aucklandiae 10 g, and white and greasy fur, added *Pogostemon cablin *10 g, Perrin 15 g, and loose stools, added yam 15 g, parched hyacinth bean 15 g, and eating little, added *Amomum villosum* (putted later) 8 g, and stomach cold pain, added Rhizoma *Corydalis* 15 g, *Evodia rutaecarpa* 8 g, and acid regurgitation, added Concha Arcae 15 g, Cuttlebone 15 g)	Clarithromycin 0.5 g bid, amoxicillin 0.5 g bid, and bismuth pectin 0.15 g qid	2	Overall effect (clinical symptoms, signs, manifestations of gastroscopy, and pathology)

Ni et al. 2013 [13]	21–66	Chronic gastritis	Deficiency cold of spleen and stomach	0.5–12	62	238 (120/118)	HQJZ + control medicine (and vomiting seriously, added dried ginger, *Pinellia*, Tangerine Peel, *Poria cocos, *and acid regurgitation, added *Coptis chinensis*, *Evodia rutaecarpa*, Cuttlebone, Concha Arcae, and stomach cold pain, seriously cold inside, vomiting, and cold limbs, added Lizhong Wan, and feeling cold, soreness, tiredness of waist and knee, added Fuzi Lizhong Wan, or added medicated leaven, *Atractylodes*, Tangerine Peel, agrimony, bitter orange, bergamot according to the symptoms)	Colloidal bismuth pectin 2 capsules tid	4	Overall effect (clinical symptoms, signs, and manifestations of gastroscopy)

Li 2013 [14]	19–76	Chronic gastritis		0.125–10	61	76 (38/38)	HQJZ (added Xiangsha Liujunzi Tang, and stomachache like needling, added Fructus Toosendan, *Spatholobus suberectus* Dunn, and gastric acid and vomiting, added *Evodia rutaecarpa*, Cuttlebone, Concha Arcae, and loose stools and not warm hands and feet, added dried ginger, Eaglewood, combined spicebush, aconite, and loss of appetite, nausea, dry and bitter mouth, and yellowish fur, added *Gardenia*, bamboo shavings, and constipation, added *Fructus Cannabis*, rhubarb, and white and damp fur, hiccup, and slow pulse, added Calyx Kaki, clove, and eructation, added bristle *Inula*, Eaglewood)	Omeprazole 20 mg bid	Intervention 2; control medicine 4	Overall effect (clinical symptoms)

Tang and Hong 2003 [15]	20–68	Chronic gastritis		0.5–>5	53	85 (43/42)	HQJZ + furazolidone 0.1 g tid (added *lanceolata* 15 g, medicated leaven 10 g, and dandelion 30 g)	Amoxicillin (if allergy, metronidazole 0.1 g) 0.5 g, furazolidone 0.1 g, and sucralfate 1.0 g, tid	3	Overall effect (clinical symptoms, manifestations of gastroscopy, and pathology, Hp)

**Table 2:** Meta-analysis and pooled results of the main outcome in included studies.

Study ID	Adverse reaction	Risk of bias	Follow-up	Random method	Overall effect OR (95% CI)	TCM syndrome effect	Hp OR (95% CI)	Gastroscope OR (95% CI)	Pathology OR (95% CI)	Overall effect *P* value
Chen and Lai 2013 [7]	Not mentioned	Low	Not mentioned	Not mentioned	3.08 (1.10, 8.62)	OR 6.67 (1.41, 31.59)				0.03

Fu et al. 2013 [8]	Not found	Low	Not mentioned	Random number table	5.21 (1.28, 21.24)	MD −5.85 (−7.71, −3.99)				0.02

Zhang 2013 [9]	Not found	Low	Not mentioned	Random number table	3.58 (0.89, 14.39)	OR 9.75 (1.16, 82.11)	6.02 (1.43, 25.40)	3.78 (1.29, 11.06)	2.83 (1.00, 7.98)	0.07

Shi 2010 [10]	Not mentioned	Low	Not mentioned	Not mentioned	3.86 (1.41, 10.57)					0.009

Li and Xu 2009 [11]	Not mentioned	Low	Not mentioned	Not mentioned	1.64 (0.51, 5.33)					0.41

L. Liu and Y. Liu 2014 [12]	Not mentioned	Low	Not mentioned	Random number table	2.62 (0.99, 6.94)					0.05

Ni et al. 2013 [13]	Not found for intervention; rash and anaphylactoid purpura of legs for 1 case in control group; increase of eosinophil for 1 case	Low	Not mentioned	Not mentioned	2.80 (1.35, 5.82)					0.006

Li 2013 [14]	In intervention group, epigastric pain, fullness, belching, and poor appetite were all less than control group	Low	Not mentioned	Not mentioned	3.62 (0.90, 14.63)					0.07

Tang and Hong 2003 [15]	Most in control group, poor appetite, upper abdominal discomfort, nausea and vomiting, and so forth; especially used metronidazole, must add metoclopramide and anisodamine, and so forth, impacted quality of lives	Low	Not mentioned	Not mentioned	1.58 (0.25, 9.95)					0.63
